# Abnormal movement of the pulmonary valve in dogs diagnosed with patent ductus arteriosus

**DOI:** 10.1007/s11259-022-10003-y

**Published:** 2023-01-12

**Authors:** Ryokichi Ishikawa, Ayaka Chen, Reie Kamatsuki, Asuka Setoguchi, Masami Uechi

**Affiliations:** 1 Ishikawa Animal Hospital, 1-14-25, Morisaki, Yokosuka, Kanagawa 238-0023 Japan; 2 JASMINE Veterinary Cardiovascular Medical Center, 1-8-37, Nakagawa, Tsuzuki, Yokohama, Kanagawa 224-0001 Japan; 3 School of Veterinary Medicine, Kitasato University, 35-1, Higashi-23bancho, Towada, Aomori 034-8628 Japan

**Keywords:** Anterior semilunar cusp, Echocardiography, Pulmonic regurgitation, Shunt flow, Surgical ligation

## Abstract

In the typical left-to-right patent ductus arteriosus (PDA), the shunt flows from the ductus arteriosus towards the pulmonary valve. Although hemodynamic changes have been carefully studied in dogs with PDA, there is very little information on the outcomes of the pulmonary valve after surgical correction of PDA. This study aimed to visualize the pulmonary valve by transthoracic echocardiography in dogs with PDA before and after surgical ligation. Prior to surgery, the movement of the anterior semilunar cusp of the pulmonary valve was obstructed by the shunted blood flow during systole in all nine dogs with PDA in this study. M-mode echocardiography revealed a continuous trajectory of the cusp, because the cusp was pushed towards the right ventricle during the whole cardiac cycle by the shunted flow. Epicardial echocardiography performed in one dog during surgical ligation of the ductus arteriosus revealed that the movement of the anterior semilunar cusp normalized immediately after ligation. B- and M-mode echocardiography may be used to support the diagnosis of PDA through observation of the pulmonary valve when color Doppler echography is not available. The findings in this study may be of importance in distinguishing PDA from PDA-mimicking diseases worth considering before the treatment process (e.g. aorticopulmonary fistulas or aberrant arteriovenous shunts).

**Supplementary Information:**

The online version contains supplementary material available at 10.1007/s11259-022-10003-y.

## Introduction

The pulmonary valve is composed of three cusps that open and close simultaneously in response to the pressure gradient of the right ventricle and the pulmonary artery (Guyton and Hall 2006). Patent ductus arteriosus (PDA) is a common congenital cardiac defect in dogs (Buchanan 2001; Orton 2003; Strickland and Oyama 2016). The ductus arteriosus connects the proximal descending aorta to the main pulmonary artery. The direction of the shunt blood flow between aorta and pulmonary artery depends on the pressure gradient, generally in a left-to-right shunt that creates a turbulent flow in the main pulmonary artery (Bussadori and Pradelli 2015; Martin and Dukes-McEwan 2010; Strickland and Oyama 2016). The diagnosis of PDA is based on auscultation of a continuous heart murmur, echocardiographic evidence of a continuous turbulent flow pattern from the aorta to the pulmonary artery by color Doppler imaging, and visualization of the ductus arteriosus by contrast angiography (Bussadori and Pradelli 2015; Oyama and Sisson 2001). 

In left-to-right PDA, there is continuous retrograde flow from the aorta into the main pulmonary artery towards the pulmonary valve. This retrograde turbulent flow directed towards the pulmonary valve may interfere with its normal function by causing prolapse of the valves (Boon 2011). Although dogs with PDA undergo careful cardiac assessments, there is little literature regarding the pulmonary valve in dogs with PDA (Boon 2011) especially after surgical intervention. This study visualizes the abnormal movement of the pulmonary valve prior to surgery and its normalization after surgery in dogs with PDA by transthoracic and epicardial echocardiography.

## Materials and methods

A prospective cross-sectional study was conducted for echocardiographic evaluation of the pulmonary valve among healthy dogs (n = 5) and dogs diagnosed with PDA (n = 9). The dogs were enrolled in the study consecutively. Healthy dogs were defined as dogs without cardiac abnormalities by auscultation, thoracic radiograph, and transthoracic echocardiography with no apparent systemic disease. PDA was diagnosed by auscultation of a continuous murmur on the left base of the heart and echocardiographic visualization of a left-to-right shunt flow through the ductus arteriosus during both systole and diastole. For comparison of transthoracic echocardiographic features of the pulmonary valve between healthy dogs and dogs diagnosed with PDA, two-dimensional, B and M-mode, and color Doppler images at the pulmonary valve level were obtained from a right parasternal short axis view in all dogs ^a^. The pulmonary valve, and the anterior semilunar cusp in particular, was compared between the two dog groups in systole and diastole. In M-mode, the cursor was placed at the tip of the anterior semilunar cusp to record the trajectory. For the dogs diagnosed with PDA, these echocardiographic images were obtained within a day before surgery and while recovering from anesthesia after the chest was closed. All dogs diagnosed with PDA in this study underwent surgical duct ligation via thoracotomy. To observe pulmonary valve movements before and after ductus arteriosus ligation, epicardial echocardiography was performed in one dog with PDA during surgery ^a^. The transducer was placed on the root of the pulmonary artery with the ultrasound beam aimed toward the pulmonary valves to include the long-axis view of the right ventricular outflow tract and main pulmonary artery by B-mode. When the anterior semilunar cusp was clearly visualized, duct ligation was performed. All echocardiographic data was obtained by a single observer.

## Results

**Fig. 1:**
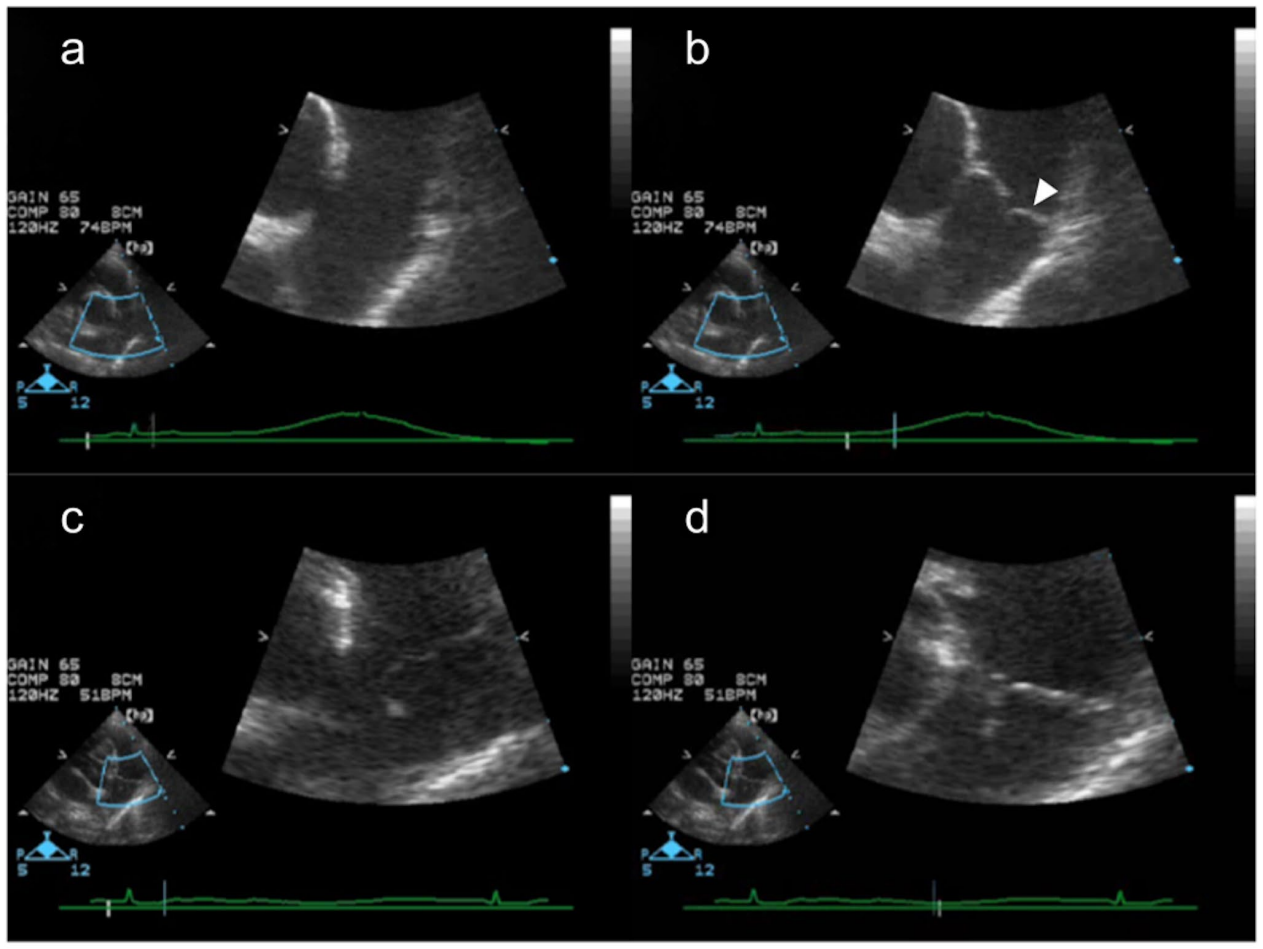
Movement of the pulmonary valve in a healthy dog and a dog with PDA Echocardiographic images of the pulmonary valve in a healthy dog (a, b) and a dog with PDA (c, d). Valves opened during systole (a) and closed during diastole (b) in healthy dogs. In dogs with PDA, the anterior semilunar cusp of the pulmonary valve did not open during systole (c). The movement of the anterior semilunar cusp during diastole was normal (d). The white arrowhead in (b) indicates the anterior semilunar cusp.

Echocardiographic data from five clinically healthy mixed-breed dogs (three males and two females, age range: 1–5 years, weight range: 5–12 kg) were obtained. These healthy dogs showed normal movement of the pulmonary valve leaflets during systole or diastole (Fig. 1a, b). To evaluate the pulmonary valve in PDA, nine dogs (three males, six females, age range: 2 months to 3 years, weight range: 1.4–10.2 kg) were enrolled in this study. The breeds included Shetland Sheepdog (n = 3), Pomeranian (n = 3), Miniature Dachshund (n = 1), Maltese (n = 1), and Chihuahua (n = 1). In B-mode echocardiography, among these dogs with PDA, none showed abnormal movement of the left semilunar cusp during systole or diastole. However, during systole, the anterior semilunar cusp failed to move simultaneously with the left semilunar cusp and did not open in all nine dogs. It was pushed toward the right ventricular cavity during all cardiac cycles and appeared elongated in comparison with the left semilunar cusp (Fig. 1c, d). In seven of the nine dogs with PDA, the edge of the anterior semilunar cusp showed a wiggly movement as the ductal flow reached the cusp in color Doppler echocardiography. Of the dogs with this wiggly movement, four had pulmonary regurgitation. 

**Fig. 2:**
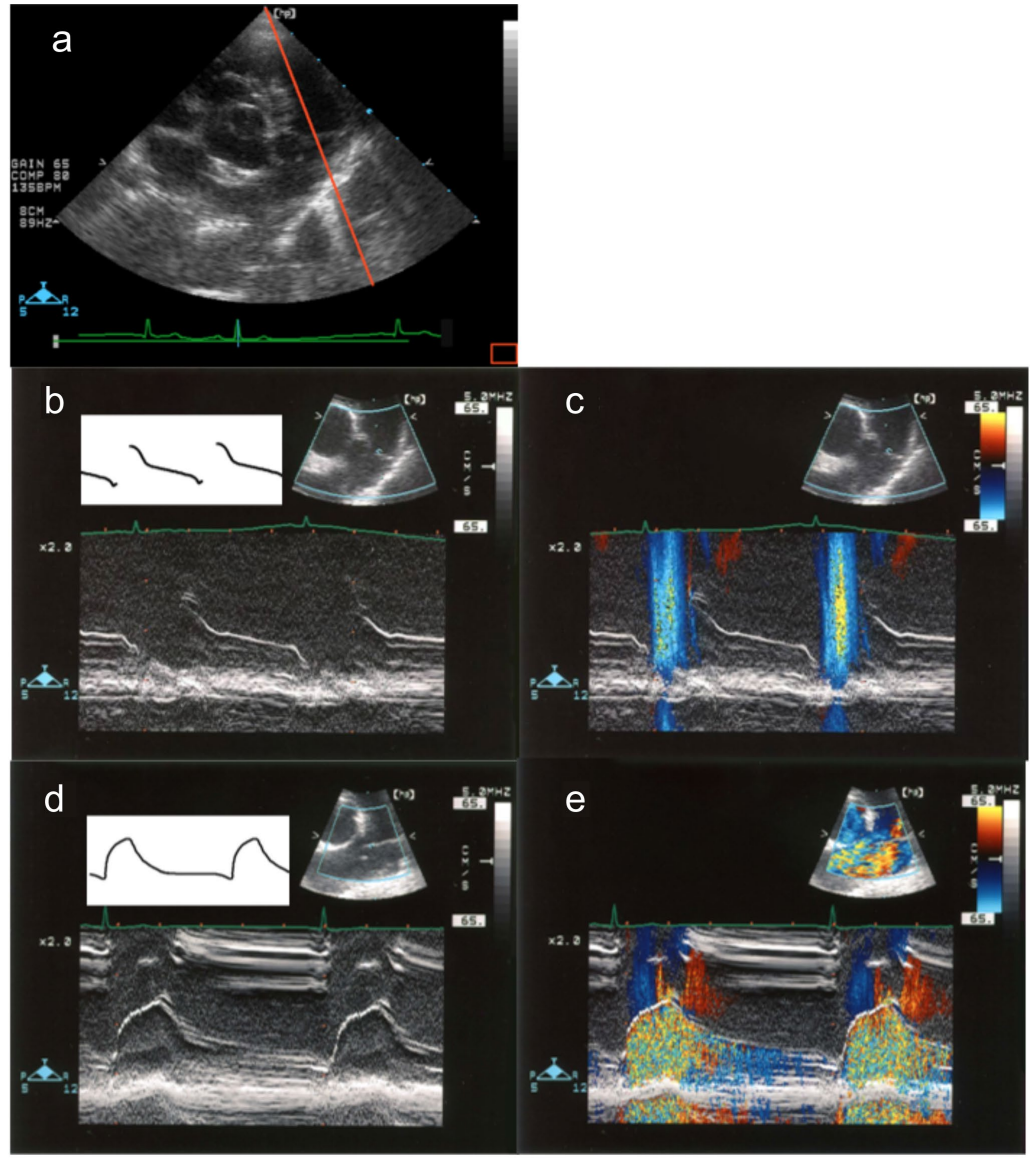
The trajectory of the pulmonary valve in a healthy dog and a dog with PDA Echocardiographic images of the trajectory of the anterior semilunar cusp with the red line in (a) resembling the cursor position in the M-mode. In a healthy dog, the trajectory was intermittent (b), and an ejection flow was observed between the trajectories by color Doppler (c). In a dog with PDA, a continuous trajectory was observed (d), and color Doppler revealed a mosaic pattern due to shunt flow on the pulmonary artery side of the cusp (e). The diagram inserted in (b) and (d) represents the trajectory pattern.

In M-mode imaging, the trajectory of the anterior semilunar cusp was intermittent in healthy dogs and continuous in all dogs with PDA (Fig. 2). Epicardial echocardiography to monitor the pulmonary valve was performed during surgery in one dog with PDA. The ductal shunt flow obstructed the movement of the anterior semilunar cusp during systole. This abnormal motion of the cusp normalized immediately after ligation of the ductus arteriosus (Online Resource 1). Complete closure of the ductus arteriosus was achieved by surgery in all nine dogs. Transthoracic echocardiography performed after the chest was closed revealed that pulmonic regurgitation, which was observed in four dogs before surgery disappeared. The anterior semilunar cusp movement was normal and no longer appeared elongated in all dogs.

## Discussion and conclusion

The pulmonary valve is composed of three cusps that open and close simultaneously in response to the pressure gradient between the right ventricle and pulmonary artery; the valve opens when the right ventricular pressure exceeds the pulmonary artery pressure, and vice versa (Guyton and Hall 2006). Pulmonic regurgitation is a common finding in dogs, but is usually mild (Rishniw and Erb 2000). In the four dogs with pulmonic regurgitation in this study, the vibration of the free end of the anterior semilunar cusp was thought to exacerbate or actually be the primary cause for pulmonic regurgitation, as it disappeared in all four dogs after PDA ligation. Not all instances of the vibrating movement of the anterior semilunar cusp were accompanied by regurgitation, suggesting that duct location or diameter may affect its presence.

In summary, the abnormal movement (the anterior semilunar cusp not opening during systole and the wiggly movement) of the pulmonary valve, which normalized after surgical intervention, was visualized by B-mode and M-mode echocardiography in dogs with PDA. This finding may be proposed as an additional hallmark of PDA, supporting the diagnosis through observation of the pulmonary valve. Whereas the diagnosis of PDA is usually not a diagnostic challenge, our finding may have particular importance for detecting dogs with aorticopulmonary fistulas or aberrant arteriovenous shunts, all mimicking PDA on auscultation or echocardiography (Leach et al. 2010; Fujii et al. 2009; Yamane et al. 2001).

Footnotes.

a S12 prove, SONOS 5500, Hewlett Packard, 12 S or 6 S prove, IE95, GE.

**Online Resource 1** Epicardial echocardiographic sectional image of the pulmonary artery at the pulmonary valve level in a dog with PDA.

Before ligation of the ductus arteriosus, the anterior semilunar cusp would not open because of the jet flow from the ductus arteriosus. The other cusp shown in the movie closes during systole. “PDA ligated” is inserted at the timepoint of ligation around 0:05. ASC, anterior semilunar cusp; MPA, main pulmonary artery; PDA, patent ductus arteriosus; RVOT, right ventricular outflow tract.

## Electronic supplementary material

Below is the link to the electronic supplementary material.

Supplementary Material 1

## Data Availability

The datasets generated in the current study are available from the corresponding author upon reasonable request.
